# Personal protective equipment for reducing the risk of COVID-19 infection among health care workers involved in emergency trauma surgery during the pandemic: An umbrella review

**DOI:** 10.1097/TA.0000000000003073

**Published:** 2021-01-12

**Authors:** Dylan P. Griswold, Andres Gempeler, Angelos Kolias, Peter J. Hutchinson, Andres M. Rubiano

**Affiliations:** From the NIHR Global Health Research Group on Neurotrauma (D.P.G., A.K., P.J.H., A.M.R.), University of Cambridge; Division of Neurosurgery (D.P.G., A.K., P.J.H.), Department of Clinical Neurosciences, Addenbrooke’s Hospital, University of Cambridge, Cambridge, United Kingdom; Clinical Research Center (A.G.), Fundación Valle del Lili, Cali; Neuroscience Institute (A.M.R.), INUB-MEDITECH Research Group, El Bosque University, Bogotá; and Neurological Surgery Service (A.M.R.), Vallesalud Clinic, Cali, Colombia.

**Keywords:** Umbrella review, broad evidence synthesis, COVID-19, global health, trauma surgery, evidence-based practice, PPE, N95 masks

## Abstract

Health care facilities in low- and middle-income countries are inadequately resourced to adhere to current COVID-19 prevention recommendations. Recommendations for surgical emergency trauma care measures need to be adequately informed by available evidence and adapt to particular settings. To inform future recommendations, we set to summarize the effects of different personal protective equipment (PPE) on the risk of COVID-19 infection in health personnel caring for trauma surgery patients.

**METHODS:**

We conducted an umbrella review using Living Overview of Evidence platform for COVID-19, which performs regular automated searches in MEDLINE, Embase, Cochrane Central Register of Controlled Trials, and more than 30 other sources. Systematic reviews of experimental and observational studies assessing the efficacy of PPE were included. Indirect evidence from other health care settings was also considered. Risk of bias was assessed with the AMSTAR II tool (Assessing the Methodological Quality of Systematic Reviews, Ottawa, ON, Canada), and the Grading of Recommendations, Assessment, Development, and Evaluation approach for grading the certainty of the evidence is reported (registered in International Prospective Register of Systematic Reviews, CRD42020198267).

**RESULTS:**

Eighteen studies that fulfilled the selection criteria were included. There is high certainty that the use of N95 respirators and surgical masks is associated with a reduced risk of COVID-19 when compared with no mask use. In moderate- to high-risk environments, N95 respirators are associated with a further reduction in risk of COVID-19 infection compared with surgical masks. Eye protection also reduces the risk of contagion in this setting. Decontamination of masks and respirators with ultraviolet germicidal irradiation, vaporous hydrogen peroxide, or dry heat is effective and does not affect PPE performance or fit.

**CONCLUSION:**

The use of PPE drastically reduces the risk of COVID-19 compared with no mask use in health care workers. N95 and equivalent respirators provide more protection than surgical masks. Decontamination and reuse appear feasible to overcome PPE shortages and enhance the allocation of limited resources. These effects are applicable to emergency trauma care and should inform future recommendations.

**LEVEL OF EVIDENCE:**

Review, level II.

## Abstract 2

Supplemental digital content is available in the text.

Many health care facilities in low- and middle-income countries are inadequately resourced. COVID-19 has the potential to decimate these already strained surgical health care services unless health systems take stringent measures to protect health care workers (HCWs) from viral exposure. A recent study showed that 15.6% of confirmed COVID-19 patients are symptomatic and that nearly half of patients with no symptoms at detection time will develop symptoms later.^1^ These factors impede and confound diagnostic triage. Improper infection prevention may create a “super-spreader” event in a high-volume health care facility or reduce available personnel. Consequently, the infection control strategy of trauma surgery staff is a top priority.

Personal protective equipment (PPE) is paramount to protect HCWs from contracting the virus and becoming disease carriers. Basic recommended PPE for trauma surgery staff of high-income country facilities include the following: (1) a surgical mask or better for all personnel interacting with patients and in the operating room (including cleaning staff), (2) N95 or better mask for all staff in close contact with the patients (<6 ft away), (3) powered air purifier respirators (PAPRs) for aerosolizing and high-risk procedures (ear, nose, throat, thoracic, and transsphenoidal neurosurgery operations); (4) universal testing of patients preoperatively to enable appropriate PPE use, and (5) changing scrubs after every procedure.^2^ These recommendations are suitable for high-resource settings but are less feasible in low-resource settings. A rapid-turnaround survey of 40 health care organizations across 15 LMICs revealed that 70% lack PPE and COVID-19 testing kits and only 65% of the respondents showed confidence in hospital staff’s knowledge about precautions to be taken to prevent COVID-19 infection among hospital personnel.^3^ Some resource-adjusted recommendations include the use of cloth masks and bandanas. While innovative, their moisture retention, reusability, and filtration are considered inferior to N95 and other masks.^4^ What is most needed are evidence-based recommendations for PPE use in LMIC surgical systems where resources are either limited or unavailable. Health care workers have been instructed to consider refraining from caring for patients in the absence of adequate PPE availability.

Evidence of the efficacy of different PPE in emergency trauma surgery setting is needed to adequately adapt recommendations of PPE use to limited resources environments. A preliminary search of International Prospective Register of Systematic Reviews, MEDLINE, the Cochrane Database of Systematic Reviews, and the JBI Database of Systematic Reviews and Implementation Reports did not find any completed or ongoing systematic reviews on the topic. The primary objective of the review was to summarize the effects of different PPE in reducing the risk of COVID-19 infection of health personnel caring for patients undergoing trauma surgery. The purpose of the review was to inform recommendations for the rational use of PPE in emergency surgery staff, particularly in low-resource environments where PPE shortages and high costs are expected to hamper the safety of HCWs and affect the care of trauma patients.

## PATIENTS AND METHODS

We conducted a broad evidence synthesis (umbrella review) to summarize the effects of PPE on the risk of COVID-19 infection in HCWs caring for patients in need of emergency surgery because of trauma. A protocol of this review following the Preferred Reporting Items for Systematic Reviews and Meta-analyses statement was registered in the International Prospective Register of Systematic Reviews (CRD42020198267). This review was conducted following the JBI methodology for systematic reviews.^5^

### Selection Criteria

#### Participants

We considered studies that included HCWs in emergency trauma surgery settings during the COVID-19 pandemic. Given the likelihood that reports on this specific population were scarce or even nonexistent, we also included studies of HCWs in any procedural and in-hospital setting, such as the operating room, the emergency department, and critical care units. Furthermore, indirect evidence from other viral respiratory diseases (especially severe acute respiratory syndrome [SARS] and Middle East respiratory syndrome [MERS]) was considered if summarized and discussed regarding the COVID-19 pandemic.

#### Intervention(s)

Different types of PPE were used while caring for patients in hospital settings (preferably in emergency surgery).

#### Comparator(s)

Comparators of interest were no PPE use and different types of PPE.

#### Outcomes

The primary outcome of interest was the risk of contagion to health personnel involved in the care of the described population during the COVID-19 pandemic, expressed as incidence, or with association measures such as risk ratios or odds ratio (OR) when compared with different PPEs or no-PPE.

#### Types of Studies

This review considered systematic reviews of experimental and observational studies, and experimental or observational studies if not included in systematic reviews that fulfilled population and intervention criteria. Only studies published in English or Spanish were included. We included preprint studies identified in our search, but no ongoing studies were considered.

#### Search Strategy

We conducted searches in the Living Overview of Evidence platform for COVID-19. The platform was consulted on July 27, 2020, using the following entries: (1) prevention or treatment, procedures, protective measures, and PPE plus population filter: COVID-19; (2) prevention or treatment, procedures, protective measures, PPE plus population filter: health workers.

#### Information Sources

The databases to be searched include the Living Overview of Evidence platform for COVID-19, a system that performs automated regular searches in PubMed, Embase, Cochrane Central Register of Controlled Trials, and more than 30 other sources. When compared with manual searches, this platform consistently identifies all the available studies associated with the terms of interest. It allows for a fast (automated) search that is easy to update, a crucial element given the urgent need to answer the research question rapidly and thoroughly.

#### Study Selection

**Figure 1:**
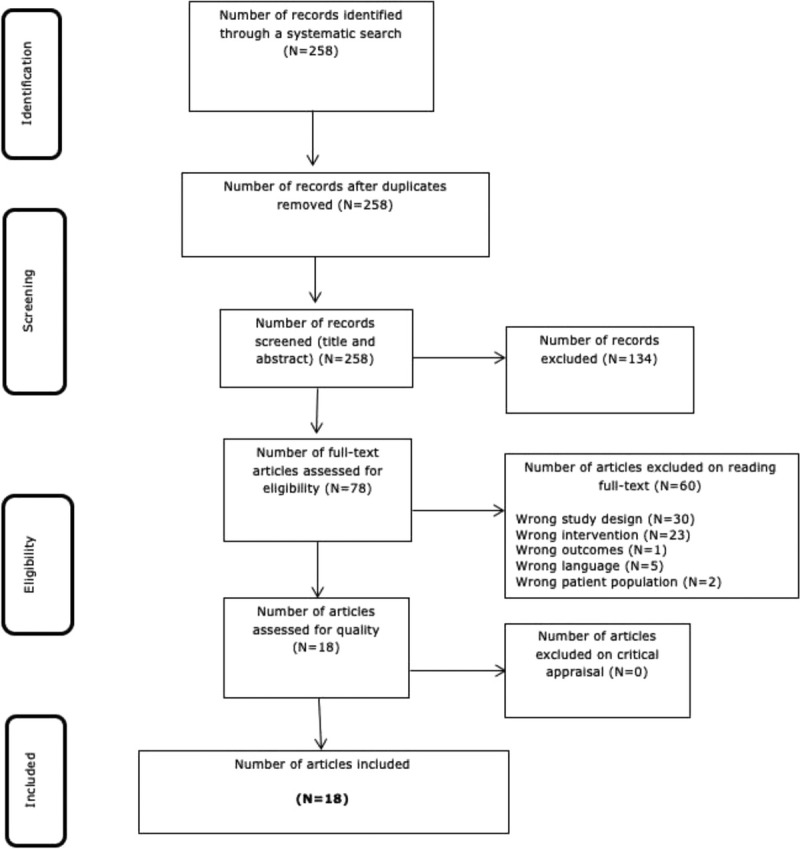
Preferred Reporting Items for Systematic Reviews and Meta-analyses search results, study selection, and inclusion process.

Following the search, all identified citations were collated and uploaded into EndNoteX9 (Clarivate Analytics, PA). The citations were then imported into JBI SUMARI for the review process. Two independent reviewers examined titles and abstracts for eligibility. Full-text review verified fulfillment of selection criteria. All decisions taken during screening were documented and are outlined in this report with a list of excluded studies. Any disagreements that arose between the reviewers were solved by consensus. The results of the search are presented in a Preferred Reporting Items for Systematic Reviews and Meta-analyses flow diagram (Fig. 1).^6^

#### Assessment of Methodological Quality

Eligible studies were critically appraised by a reviewer and verified by a second reviewer using the AMSTAR tool (Assessing the Methodological Quality of Systematic Reviews, Ottawa, ON, Canada). The risk of bias was assessed for only the primary outcome: infection of HCWs by COVID-19 or similar. The results of the critical appraisal are reported narratively and are considered for discussion of results.

All included studies, regardless of their risk of bias, underwent data extraction and synthesis.

#### Data Extraction

Data were extracted from the included studies by a reviewer and verified by a second reviewer using a data extraction tool from JBI SUMARI.^5^

The data extracted include specific details about the populations, study methods, interventions, and outcomes of significance to the review question and specific objectives. Disagreements were solved by consensus.

#### Data Synthesis

Studies were summarized narratively considering their scope, number of included studies, and risk of bias. Effect sizes from systematic reviews and individual studies not included in them are expressed as ORs (for dichotomous data) with their 95% confidence intervals (CIs).

#### Assessing Certainty in the Findings

The Grading of Recommendations, Assessment, Development, and Evaluation approach for grading the certainty of the evidence was followed. Grading the certainty of the evidence was not undertaken if adaptation from the identified reviews using the Grading of Recommendations, Assessment, Development, and Evaluation approach was considered complete and adequate.^7,8^ The certainty of the evidence was considered for interpretation and discussion of findings.

## RESULTS

### Study Inclusion

The study selection process is illustrated in Figure 1.^6^ The described search identified a total of 258 records. After title and abstract screening, 78 studies were considered for full-text review, of which 60 were excluded. Reasons for exclusion were as follows: wrong study design (n = 30),^9–38^ wrong intervention (n = 23),^39–60^ wrong outcomes (n = 1),^61^ wrong language (n = 5),^62–66^ and wrong patient population (n = 2).^67,68^ This left 18 studies for appraisal, extraction, and synthesis.^4,69–84^

Appendix I (http://links.lww.com/TA/B877) shows a list of the 60 excluded studies, with reasons for their exclusion. Studies excluded because of wrong study design were mostly narrative reviews, guideline recommendations, and case reports. Wrong setting was the reason for exclusion when studies were developed in a nonhospital or community setting. Studies that did not report data related to COVID-19 contagion in HCWs were also excluded.

### Included Studies

Seventeen of the 18 included studies were systematic reviews,^4,69–72,74,75,77–81,83,85^ and 1 was a qualitative evidence synthesis.^83^ Appendix II (http://links.lww.com/TA/B878) provides details of the characteristics of the included studies. All but one study was published in 2020.^77^ Data extracted from reviews included thousands of participants from 35 different countries.

Ten of 17 systematic reviews evaluated the risk of contagion for respiratory viral infections,^4,33,70–72,76,80,82^ of which 6 included outcome data for COVID-19 infection.^4,70,74,76,80,82,84^

Four systematic reviews^71,74,77,79^ evaluate other respiratory pathogens such as seasonal influenza, SARS, H1N1, and MERS.

### Methodological Quality

Tables 1 and 2 show the results of the risk of bias assessment of the included studies. Overall, the methodological quality of the 18 included studies was assessed as moderate to high by JBI appraisal standards, and no disagreements occurred between the reviewers. Of the 17 included systematic reviews, 9 had low risk of bias overall (fulfilled all indicators),^70,71,74–77,79,80,82^ and 5 had concerns in 1 indicator,^69^ because of not performing risk of bias assessment. Two reviews had risk of bias in more domains,^72,84^ having no method of study appraisal and no method for minimizing errors in data extraction, and failing to report a risk of bias assessment. One review only fulfilled four indicators^83^; in addition to failing in the aforementioned indicators, it lacked future directives and recommendations for policy and clinical practice. All systematic reviews clearly stated the review question, applied appropriate inclusion criteria, and search strategy. Several reviews chose not to combine studies for meta-analysis because of study limitations and heterogeneity in study designs, comparisons, and analyses. Our search also found a qualitative research synthesis with low risk of bias overall.^83^

**TABLE 1:** Qualitative Research

Citation	Q1	Q2	Q3	Q4	Q5	Q6	Q7	Q8	Q9	Q10
Houghton et al.,^73^ 2020	Y	Y	Y	Y	Y	Y	Y	Y	Y	Y
%	100.0	100.0	100.0	100.0	100.0	100.0	100.0	100.0	100.0	100.0

**TABLE 2:** Systematic Reviews

Citation	Q1	Q2	Q3	Q4	Q5	Q6	Q7	Q8	Q9	Q10	Q11
Abdelrahman et al.,^69^ 2020	Y	Y	Y	Y	Y	U	Y	Y	N	Y	Y
Ana et al.,^70^ 2020	Y	Y	Y	Y	Y	Y	Y	Y	Y	Y	Y
Bartoszko et al.,^71^ 2020	Y	Y	Y	Y	Y	Y	Y	Y	Y	Y	Y
Carl-Etienne et al.,^72^ 2020	Y	Y	Y	Y	N	N	N	N/A	N/A	Y	Y
Chou et al.,^4^ 2020	Y	Y	Y	Y	Y	Y	Y	N/A	N/A	Y	Y
Chu et al.,^82^ 2020	Y	Y	Y	Y	Y	Y	Y	Y	Y	Y	Y
Toomey et al.,^85^ 2020	Y	Y	Y	Y	Y	Y	Y	N/A	N/A	Y	Y
Fouladi et al.,^83^ 2020	Y	Y	Y	Y	N/A	N/A	N/A	N/A	N/A	N	N/A
Iannone et al.,^74^ 2020	Y	Y	Y	Y	Y	Y	Y	Y	Y	Y	Y
O’Hearn et al.,^75^ 2020	Y	Y	Y	Y	Y	Y	Y	Y	Y	Y	Y
Liang et al.,^76^ 2020	Y	Y	Y	Y	Y	Y	Y	Y	Y	Y	Y
MacIntyre and Chughtai,^84^ 2020	Y	Y	Y	Y	N	N/A	N	N/A	N/A	Y	Y
Offeddu et al.,^77^ 2017	Y	Y	Y	Y	Y	Y	Y	Y	Y	Y	Y
Prashanth et al.,^78^ 2020	Y	Y	Y	Y	Y	Y	Y	N/A	N/A	Y	Y
Tom et al.,^79^ 2020	Y	Y	Y	Y	Y	Y	Y	Y	Y	Y	Y
Verbeek et al.,^80^ 2020	Y	Y	Y	Y	Y	Y	Y	Y	Y	Y	Y
Zorko et al.,^81^ 2020	Y	Y	Y	Y	Y	Y	Y	N/A	N/A	Y	Y
%	100.0	100.0	100.0	100.0	82.35	76.47	82.35	58.82	52.94	94.11	94.11

N, no; Q, question; U, unclear; Y, yes.

### Review Findings

We did not identify comparative studies of PPE effect on the risk of COVID-19 contagion in the emergency surgery setting. We did identify systematic reviews of observational studies of COVID-19 in HCW as well as of experimental and observational studies that also addressed this question in HCWs regarding other coronavirus epidemics (SARS and MERS epidemics) considered generalizable to the COVID-19 pandemic. Some studies also assessed and summarized evidence from other viral respiratory illnesses, such as H1N1 or influenza, and reported results consistent with those of the coronaviruses outbreaks.

A high-quality systematic review that evaluated the effect of physical distancing face masks and eye protection on preventing COVID-19 contagion included 172 studies, considering evidence from COVID-19, MERS, and SARS.^82^ Regarding the use of eye protection, pooled analysis of 13 unadjusted and 2 adjusted studies suggested a reduced risk of contagion with eye protection compared with no eye protection (unadjusted respiratory rate [RR], 0.34; 95% CI, 0.22–0.52; adjusted OR, 0.22; 95% CI, 0.12–0.39). Regarding masks, the authors identified 30 comparative studies that focused on the effect of different masks and respirators on virus transmission in HCWs or patients and 13 studies that addressed the same effect for eye protection. They report that the use of a surgical mask compared with no face mask was associated with a considerable reduction in risk of contagion (OR, 0.33; 95% CI, 0.17–0.61). An even larger effect was seen when comparing N95 and N95 equivalent respirators to no mask (OR, 0.04; 95% CI, 0.004–0.30). Such estimates are based on studies, including a total of 12,817 participants. Adjusted and unadjusted studies were considered, and both estimates were consistent with the mentioned effect on contagion risk reduction when considering N95 or surgical/medical masks versus no mask (adjusted OR, 0.15 (0.07–0.34); unadjusted RR, 0.34 (95% CI, 0.26–0.45). Evidence for the precisely estimated reduction was rated as low by the authors, given some inconsistency and risk of bias. Nevertheless, the beneficial effect of mask protection was large, and they considered it of high certainty.^82^ They report that N95 had a stronger protective association compared with surgical masks or 12- to 16-layer cotton masks and both N95 and surgical masks also had a stronger association with protection versus single-layer masks. This review was considered pivotal because of the high number of included studies, the recent date of publication, and the adequacy of methods and reporting. Challenges reported in the studies included frequent discomfort, high resource use, less clear communication, and perceived reduced empathy of care providers by their patients.^82^

A rapid systematic review that also addressed the effect of masks to prevent COVID-19 infection considered evidence from the current pandemic in addition to the SARS and MERS epidemics.^4^ The review reports a reduction of risk of transmission associated with the use of masks in general. It suggests a more significant reduction associated with N95 respirators compared with surgical masks in the hospital setting (an effect seen for COVID-19 independently, as well as with the other coronaviruses outbreaks).

Other reviews considered evidence from viral respiratory illnesses, including influenza or H1N1, and report a beneficial effect of PPE (medical masks or N95 respirators) on contagion risk reduction.^71,74,76,77,79,84^ One of these reviews reports that the use of masks by HCWs and non-HCWs can reduce the risk of respiratory virus infection by 80% compared with no-mask (OR, 0.20; 95% CI, 0.11–0.37).^76^ Furthermore, respirators were found to be more protective than surgical masks, and surgical masks more protective than cloth masks.^84^ There appears to be no difference between respirators and medical masks when used in non–aerosol-generating procedures low-risk environments.^71,79^ Conversely, no significant evidence was found that supported an equivalence claim of medical masks with respirators in their level of protection against COVID-19 or other similar viruses.^78^ In moderate- and high-risk hospital settings, N95 is associated with more significant reductions in risk of contagion.^4,84^

A systematic review based on experimental designs only found that N95 respirators halve the risk of any respiratory illness compared with surgical masks; the certainty of the evidence was low because of baseline differences, indirectness of evidence for COVID-19, and low event rates that account for imprecision.^74^ The reduction in contagion risk calculated from 2 RCTs was estimated to be as follows: RR, 0.43; 95% CI, 0.29 to 0.64; and *I*^2^ = 0%, from pooled analysis, with an absolute effect of preventing 73 (95% CI, 91–46) more infections per 1000 HCWs wearing N95 respirators compared with surgical masks.^74^

Among the included studies, one reported on the use of PAPRs.^70^ Based on observational studies, the authors report that they did not find a difference in risk of contagion in HCWs when comparing PAPR devices with other more compliant protective elements (N95, FFP2). They found that PAPR users reported higher heat tolerance but limited mobility and reduced audibility.

Regarding decontamination, a systematic review assessed the effectiveness of ultraviolet germicidal irradiation for the decontamination of PPE and its impact on PPE performance.^75^ Its findings support that the use of a cumulative ultraviolet-C dose of at least 40,000 J/m^2^ results in adequate decontamination without affecting performance or fit afterward. Another review on the subject reported that mask (N95) performance was best conserved using dry heat decontamination and that vaporous hydrogen peroxide and ultraviolet germicidal irradiation are effective decontaminants. However, its effect on surgical masks is unknown.^81^ The authors also state that bleach is not safe for decontamination since it alters mask performance and might be associated with health risk for users.

A qualitative evidence synthesis that searched for barriers and facilitators of HCWs adherence to PPE protocols included 20 studies of moderate- to high-quality overall (10 from Asia, 4 from Africa, 4 from Central and North America, and 2 from Australia).^73^ They report that HCWs were unsure to follow recommendations when they are long and ambiguous or do not reflect national or international guidelines. Some were overwhelmed because of constantly changing guidelines and by the increased workload and fatigue associated with PPE use because of preparation and cleaning. A serious concern was the lack of PPE or the low quality of the available items, pointing at a need to adjust supplies during the pandemic. Health care workers reported that it was challenging to use masks and other equipment when it made patients feel isolated, frightened, or stigmatized. Of course, discomfort associated with wearing PPE was also reported.

## DISCUSSION

Our review aimed at summarizing the available evidence of the effect of different PPE on the risk of COVID-19 infection among HCWs caring for patients requiring urgent trauma assessment and surgical care. We did not find experimental studies that assessed PPE on emergency trauma surgery settings during the pandemic. Limited observational evidence from COVID-19, indirect evidence from other health care settings, and other viral outbreaks were all considered to answer our research question given that the population (HCW) and intervention (PPE) of interest were the same and thus considered applicable to the emergency surgery setting.

The available evidence was consistent to show that the use of N95 respirators and surgical masks is associated with a reduced risk of coronaviruses respiratory illness compared with no mask use, with high certainty on this beneficial effect.^4,82^ In moderate- to high-risk environments, especially in aerosol-generating procedures, evidence suggests that N95 respirators are associated with a more significant reduction in risk of COVID-19 infection compared with surgical masks, an effect seen in observational COVID-19 studies and experimental viral respiratory illness studies. Low-quality evidence estimates from these studies suggest a relative reduction of 50% in the risk of contagion associated with N95 respirators compared with surgical masks. Eye protection also significantly reduces the risk of contagion compared with no-eye protection. Furthermore, the decontamination of masks and respirators with ultraviolet germicidal irradiation, vaporous hydrogen peroxide, or dry heat is effective and does not affect PPE performance or fit. This evidence should inform decontamination and reuse protocols to avoid shortages and enhance resource allocation and use. The costs associated with additional protective measures during the COVID-19 pandemic could be significant and affect health care institutions in low- and middle-income countries. The cost-effectiveness of interventions must also be taken into consideration to generate recommendations during the current pandemic. The possibility to decontaminate and reuse different types of masks can be determined in shortages and will probably reduce costs without affecting HCW’s safety.

In a survey of 5,442 neurosurgical staff members in Hubei province, among 120 participants that were infected, 78.3% reported wearing surgical masks and 20.8% failed to use any protection when exposed to the source of infection. A total of 1,287 operated under level 2 protection, and only 1 was infected,^28^ further illustrating the pertinence of wearing adequate PPE when caring for surgical trauma patients.

Expert recommendations developed from a study of emergency tracheal intubation in 202 patients with COVID-19 in Wuhan, China, notes that, while PAPRs were the PPE of choice when face shields or full hoods without PAPR were substituted, there were no instances of infection of operators.^10^ Because the risk of virus exposure due to self-contamination is high during the removal of PPE, educational training for proper donning and doffing of PPE as well as monitoring for compliance is essential. The minimum recommended PPE is eye protection, a fit-tested respirator (N95 or FFP3), a fluid-resistant gown, and gloves. The French guidelines recommend FFP2-type protective filtering masks when performing any aerosol-generating procedures.^12^ Guidelines for chest compressions recommend level 3 PPE, which includes an FFP; disposable fluid-resistant gown, disposable apron, and gloves; fluid-resistant surgical mask; and eye or full-face protection.^17^ Recommendations for otolaryngologists include fluid-resistant FFP3/N95 mask, disposable and fluid resistant gloves, and gown, glasses, or a full face shield.^14^

Intubation of trauma patients is a high-risk-of-contagion procedure during the COVID-19 pandemic. A survey in 503 hospitals from 17 countries included 1,718 HCWs performing 5,148 tracheal intubations and measured a 10.7% incidence of COVID-19 infection after tracheal intubation.^30^ Most participants reported wearing gloves, gown, eye protection, and FFP2/FFP3/N95/N100 respirators. Simulation studies have assessed the effect of additional protective and preventive measures, such as transparent plastic boxes or PAPR, on the vision, comfort, and success of tracheal intubations.^33,34^

A survey of HCWs realities and perceptions during the pandemic in Latin America included 936 participants and reported low access to disposable gowns (67.3%), N95 respirators (56.1%), and facial protective shields (32.6%). Even access to disposable surgical masks was reported by only 83.9% of participants.^31^ This emphasizes the need for rational use of limited PPE during the pandemic in LMICs to ensure HCW safety without withholding urgent trauma care.

Our findings regarding decontamination should be considered as a feasible solution for the limited access to N95 equivalent respirators during shortages and in limited resources environments. Also, to avoid such shortages, it appears that N95 respirator equivalents use should be limited to moderate- to high-risk environments, when caring for patients with confirmed COVID-19, or for suspicious or unknown status patients who need emergency surgery because of trauma.

## STRENGTHS AND LIMITATIONS

Our review used an automated search platform where evidence on COVID-19 is available. This strategy streamlined the rapid nature of the review while ensuring that all relevant studies were identified. Using an automated system has the additional long-term advantage of facilitating review updates by quickly identifying new studies that satisfy selection criteria. Our review also has the strength of having critically assessed all included studies. We report on the estimates and evidence grading of the identified high-quality systematic reviews. A metanalysis of systematic reviews results was not planned in our review protocol and was not considered adequate, given the overlap of included studies among reviews and the variation in selection criteria. Despite the differences between reviews, the consistency of findings among the reviews provides high certainty of the beneficial effects of PPE in the hospital setting. The main limitation of our review is that evidence from our specific setting of interest, that is, emergency trauma surgery, was not identified. Nevertheless, extrapolation from other clinical settings such as the emergency room, COVID-19 wards, and critical care during the pandemic was considered adequate given the characteristics of the intervention and the similarities to the setting of interest.

## CONCLUSIONS

The use of PPE drastically reduces the risk of COVID-19 compared with no mask use in HCWs in the hospital setting. Respirators like N95 or equivalent provided more protection and were found to halve the risk of COVID-19 contagion in HCWs from moderate- and high-risk settings. Eye protection also provides additional protection and is associated with reduced incidence of contagion. These effects apply to emergency trauma care. Decontamination and reuse appear as feasible measures that could help overcome PPE shortages and enhance the allocation of limited resources.

### Recommendations for Practice

When caring for a trauma patient with suspected or unknown COVID-19 status, HCWs should use at least N95 respirators or equivalents to reduce the risk of COVID-19 infection adequately. Decontamination with ultraviolet light, hydrogen peroxide, and dry heat should be made available.

### Recommendations for Research

**Figure 2:**
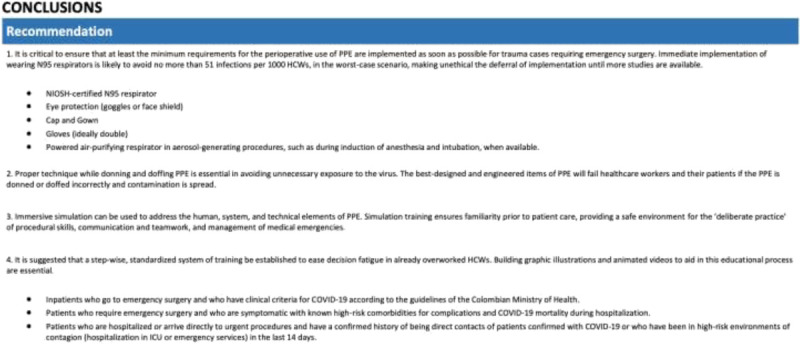
Proposed general recommendations.

Robust RCTs comparing the efficacy of surgical masks versus N95 respirators in HCWs caring for trauma patients are potentially unethical, as existing data show a significant protective effect, thus requiring emergency trauma surgery staff to wear N95 respirators when available. As of October 2020, there is a lack of consensus among international experts surrounding the topic of aerosol transmission, meaning that viral microdroplets are capable of floating in the air without being pulled down by gravity. This means that, if someone coughs, sings, or even breathes, the microdroplets can stay in stagnant air for up to 16 hours and, with normal ventilation, between 20 minutes and 4 hours. While multiple studies have discussed how SARS-coronavirus 2 can be found in aerosols, including one from May and another from April,^86,87^ a group of epidemiologists in late July characterized research on aerosol transmission as unconvincing and cited extensive published evidence from across the globe showing that the overwhelming majority of viral spread is via large respiratory droplets.^88^ The Centers for Disease Control did not acknowledge aerosol transmission as an important route for viral transmission until September 2020, placing aerosol ahead of droplet transmission as the predominant mechanism of viral spread. However, just a few days later, the statement was recalled, with updated guidelines saying HCWs need an N95 respirator for aerosol-generating procedures, only.^89^ The hospital administrators and epidemiologists who argue that the virus is mainly droplet spread claim N95 respirators and strict patient isolation practices are not necessary for routine care of COVID-19 patients. It is essential to develop a complete understanding of the transmissibility of SARS-coronavirus 2 because it drives two different sets of protective practices, touching on everything from airflow within hospital wards to patient isolation to choices of PPE. Enhanced protections would be expensive and disruptive and would have strong implications on costs, especially in low-resources environments. Amid the uncertainty, adopting the highest possible forms of protection seems the best course of action (Figure 2).
